# Supplementary material for: Academic resilience in European countries: The role of teachers, families, and student profiles
Source: PLoS One. 2021 Jul 2;16(7):e0253409. doi: 10.1371/journal.pone.0253409 (PMC8253434; doi:10.1371/journal.pone.0253409)
Supplement: Attachment — Submitted filename: 04_Response to Reviewers.docx [file pone.0253409.s001.docx]

Reviewer #1: Dear authors,

Thank you very much for your contribution. I have enjoyed reading this paper very much. I particularly appreciate the robustness of the data used and the multilevel approach to provide meaningful interpretations of the results. The introduction is also very clear signaling the importance of identifying potential moderators of academic resilience across countries.

Thank you very much for your assessment.

As pointed out by the authors, the results are aligned with expectations. It is indeed not surprising that confidence in reading was the variable most strongly linked to academic resilience. Self-perception of competence and reading performance are mutually reinforcing. So when higher-performing students receive and process performance feedback, their perception of competence tends to be higher. Since academic resilient students are defined as students in the top quartile of reading comprehension, it would be reasonable to expect that they would have higher confidence in reading. It would be good to explain this association further when discussing the results (see Marsh and Craven, (2006)).

The suggestion that academic self-concept and achievement mutually reinforce each other, and each one drives gains in the other, seems correct, and relevant to our study. Hence we have added to our discussion of the results, including citing Marsh and Craven (2006). This is highlighted via the change tracking in the new version.

Furthermore, this would likely impact most if not all, student-level variables. To some extent, it may explain why student-level variables are comparatively stronger in magnitude than teacher-level variables. Therefore, I would suggest considering to use grade repetition (or past grades if available) as a proxy to control for past achievement. Otherwise, it might good to point this out in the limitations of the study. The consequences of comparing the strengths of students and family variables with variables related to teaching are important from a policy perspective, as students and family variables are usually under a softer influence from education policies than teaching practices.

Unfortunately, PIRLS does not collect data on grade repetition, nor is it a simple matter to recode it from students birth dates given the variety of education systems. Lacking this measure is a limitation that prevents this information from being used to monitor prior achievement. For this reason, we have added this suggestion to the study limitations.

Finally, I would suggest considering to add summary tables/figures to help to visualize the results. For example, the average beta/odds ratio (and SD) per variable, percentage of countries where the coefficient was significant, EU average results, etc. I would also suggest considering adding (or citing) the percentage of resilient students by countries and the correlation matrix of the models' variables.

Following the reviewer’s suggestion, we have added the following tables: *Index of Economic, Social and Cultural Status, and percentage of resilient students for the European Union countries; Correlations of student and family contextual variables;* *Correlations of variables related to teaching;* *Percentage of countries where the coefficient was significant for student and family contextual variables and Percentage of countries where the coefficient was significant for variables related to teaching*. In the new version, added using change tracking, they appear as the renumbered Tables 2, 3, 4, 7, and 8.

Response to reviewer 2.

Reviewer #2: The paper “Academic Resilience in European countries: The role of Teachers, Families, and Student profiles” addresses a relevant topic which contributed to improve educational policies. I think the paper is well-written and clear, although I missed the authors described more details about the results. I just have some minor comments.

Thank you for your assessment. We respond to each of your comments below.

The literature review clearly exposes the relevance of the study based on previous researches. However, there is just a short reference to the influence of teachers´ training, and it is relevant to understand why the authors include them in the model and also to help in the discussion of the results. I would add some information about how the teachers´ training could affect the students´ academic resilience. It is clear why the teachers´ variables related to academic practices are included, but how is the training related to resilience? More evidence needed.

In the introduction, highlighted via change tracking, we have added references that explain the importance of teachers taking part in professional development activities for improving their students’ performance and capacities for resilience..

In table 1, there is a column for the number of home questionnaires. It is not clear why there is not a home questionnaire for all the students. It should be clarified in page 7 (around line 119) in order to help understanding the data.

Thank you for the suggestion. We have explained why there is not a family questionnaire for all students on line 141.

In page 9, the authors describe all the variables used. However, there are some where the details are not complete, such as “engaged in reading lessons”, “like reading” and “early literacy activities before school”. It also happens with some variables related to family and teachers. I think the information about the variables should be consistent. Either including all the variables or not doing that, but doing the same thing in all the cases. It could be easier to include a table where all the variables are deeply explained. It would make that part less repetitive.

In the methodology section we have completed the descrition of the variables used in the study.

After tables 2 and 3 the readers would expect a description of the results. All the results are included in the discussion section but it would be illustrative to summarize main results in the results section.

This is a good suggestion: we have added Tables 7 and 8 which summarize the results, presenting the most important for both the individual and school settings.

In the discussion section, I missed a discussion about potential patterns found between countries. The authors point to cultural and social elements as responsible of some of the results but: are similar countries reaching the same results? Are countries with similar characteristics having the same variables affecting the academic resilience?

This is an interesting question. Analyzing the data in detail, we were not able to detect patterns by country, and therefore we did not mention them. However, it is true that there is a broad range of education systems, which associated with different cultures and sensibilities, makes generalized patterns very unlikely.

In relation to the teachers training, is it related to the resilience because the topics of the training are directly related to the topic measured as performance in students?

We have added a clarification in the conclusions about the relationship between complementary training and its impact on resilience. The addition is highlighted in track changes.

Other minor issues:
- Page 14, line 272: the expression “may vey well be biased” is not clear to me.

We have clarified that it refers to an underestimation of standard errors that may produce significance where there is none in reality.

- Page 15, line 287- significance level should be at 1% instead of at 10%.

The 10% is correct, we have changed the order to avoid confusion.

- In table 2 you use “sex” and in table 3 you use “gender”. Maybe you can unify the use of the term as I assume you are referring to the same thing in both cases.

Thank you. We have chosen to use “gender” in both cases to avoid inconsistencies.
